# Recovery From Form-Deprivation Myopia in Chicks Is Dependent Upon the Fullness and Correlated Color Temperature of the Light Spectrum

**DOI:** 10.1167/iovs.63.2.16

**Published:** 2022-02-08

**Authors:** Arumugam R. Muralidharan, Shermaine W. Y. Low, Yong Chong Lee, Veluchamy A. Barathi, Seang-Mei Saw, Dan Milea, Raymond P. Najjar

**Affiliations:** 1 Singapore Eye Research Institute, Singapore; 2 Ophthalmology and Visual Sciences Academic Clinical Programme, Duke-NUS Medical School, Singapore; 3 Department of Ophthalmology, Yong Loo Lin School of Medicine, National University of Singapore, Singapore; 4 Saw Swee Hock School of Public Health, National University of Singapore, Singapore; 5 Singapore National Eye Centre, Singapore

**Keywords:** myopia, full-spectrum light, correlated color temperature, axial length, sclera, choroid, animal models

## Abstract

**Purpose:**

The purpose of this study was to evaluate the impact of full-spectrum light-emitting diodes mimicking sunlight (Sunlike LEDs) on ocular growth and refractive error development in a chicken model of myopia.

**Methods:**

One-day old chicks (*n* = 39) were distributed into 3 groups and raised for 28 days in isoluminant (approximately 285 lux) fluorescent (*n* = 18, [FL-4000], correlated color temperature [CCT] = 4000 K) or Sunlike LED (*n* = 12, [SL-4000], CCT = 4000 K; *n* = 9, [SL-6500], CCT = 6500 K) white lighting environments. Form-deprivation myopia was induced monocularly from day 1 post-hatching (D1) until D14. On D14, form deprivation was halted and the recovery of form-deprived (FD) eyes was monitored until D28. Axial length (AL), refraction, choroidal thickness, and anterior chamber depth were measured in vivo on D1, D7, D14, D22, and D28. Differences in outcome measures between eyes and groups were compared using 2-way repeated-measures ANOVA.

**Results:**

AL and myopic refraction of FD eyes increased similarly among groups during form-deprivation. FD eyes of animals raised under SL-4000 (D22: *P* < 0.001 and D28: *P* < 0.001) and SL-6500 (D22: *P* = 0.006 and D28: *P* < 0.001) recovered faster from axial elongation compared with animals raised under FL-4000. The refractive status of FD eyes reared under SL-6500, not under FL-4000 or SL-4000, was similar to control eyes on D28 (*P* > 0.05). However, SL-4000 and SL-6500 exhibited similar refraction on D28 than FL-4000 (*P* > 0.05). Choroidal thickness was significantly greater in FD eyes of chickens raised under SL-6500 than in animals raised under FL-4000 (*P* = 0.03).

**Conclusions:**

Compared to fluorescent light, moderate intensities of full-spectrum Sunlike LEDs can accelerate recovery from form-deprivation myopia in chickens, potentially through a change in the choroid-mediated pathway.

Myopia is due to a mismatch between the axial length (AL) of the eye and the focusing power of its optics during growth, this causes images of distant objects to be focused in front of the retina and appear blurred.^1^ The prevalence of myopia is rising, and it is estimated that almost 50% of the world's population will be myopic by 2050, making this condition an even greater socio-economic burden.^2^^,^^3^ Although blurred vision can be corrected with glasses, lenses, or refractive surgery, there are still risks of blindness from pathologies associated with high myopia.^4^

With the strong need to delay myopia onset or slow its progression, several approaches have been proposed, including increasing time spent outdoors by children.^5^ In fact, children who spend more time outdoors have a lower risk of developing myopia and experience a reduction in myopia progression.^6^^–^^10^ The protective effects of outdoor exposure are independent of physical activity,^11^ and may be, at least in part, due to the high levels and spectrotemporal characteristics of sunlight, which are suboptimal in indoor lighting.^12^

The direct illumination from the sun at noon can rise above 130,000 lux, whereas shaded areas outdoors can range from 15,000 to 25,000 lux.^13^ Recently, lower levels of light, from 5556 to 7876 lux, have also been found in the shade under trees, whereas an open-field light intensity during different times of day was reported to range from 11,080 to 18,176 lux in cloudy conditions.^14^ In comparison, indoor illumination usually ranges between 100 and 500 lux.^13^ The spectral composition of sunlight also differs from that of artificial indoor lighting. Sunlight has a full spectrum that includes wavelengths ranging from approximately 300 nm to approximately 1200 nm, whereas standard fluorescent (FL) indoor lights emit wavelengths ranging from approximately 400 nm to approximately 700 nm in a spiked distribution, peaking in blue, green, and red.^15^ Furthermore, the correlated color temperature (CCT) of sunlight is dynamic throughout the day, ranging from approximately 2000 K at sunrise or sunset to over 10,000 K on a clear blue sky at midday.

The importance of the spectral composition of light on axial ocular growth and myopia development has been demonstrated in several animal models.^16^^,^^17^ For instance, chickens^16^ and guinea pigs^18^ raised in longer wavelengths of light are more susceptible to ocular axial elongation and increased myopia, whereas exposure to monochromatic short-wavelength blue lights either induces hyperopia or protects against myopia development, in chicks,^17^ guinea pigs,^19^ and some rhesus monkeys.^20^ Conversely, exposure to long wavelength monochromatic lights has also been reported to be protective against myopia in rhesus monkeys.^21^ To date, the impact of CCT and spectral distribution of artificial white light on emmetropization and refractive error development remains under-investigated. Nevertheless, in an interventional study not focused on the spectral composition of light, Hua et al.^22^ reported that fluorescent light with a CCT of 6500 K, at ambient light levels of 558 lux, could decrease myopia onset, as well as reduce axial growth among both myopic and non-myopic school children aged 6 to 14 years. In addition, our group has recently shown that compared to standard light-emitting diode (LED) white light (CCT = 3900 K), blue-enriched white light (CCT = 9700 K) can slow axial elongation induced by form-deprivation myopia (FDM), and accelerate recovery from FDM in a chicken model. Early studies by Wallman and Adams demonstrated that recovery from FDM in chicks involves visual feedback in regulating emmetropization,^23^ and, according to Troilo et al.,^24^ recovery from FDM is considered as one of the key indicators of the visual regulation of refractive development. Therefore, studying the recovery from FDM provides insights into signals associated with emmetropization, ocular growth, and refractive development.

In this study, we evaluated the impact of moderate intensities of LEDs with two different CCTs having full sunlight-like emission spectra on ocular growth, and myopia development and recovery in a chicken model of FDM.

## Methods

### Animals and Experimental Paradigms

**Figure 1.**
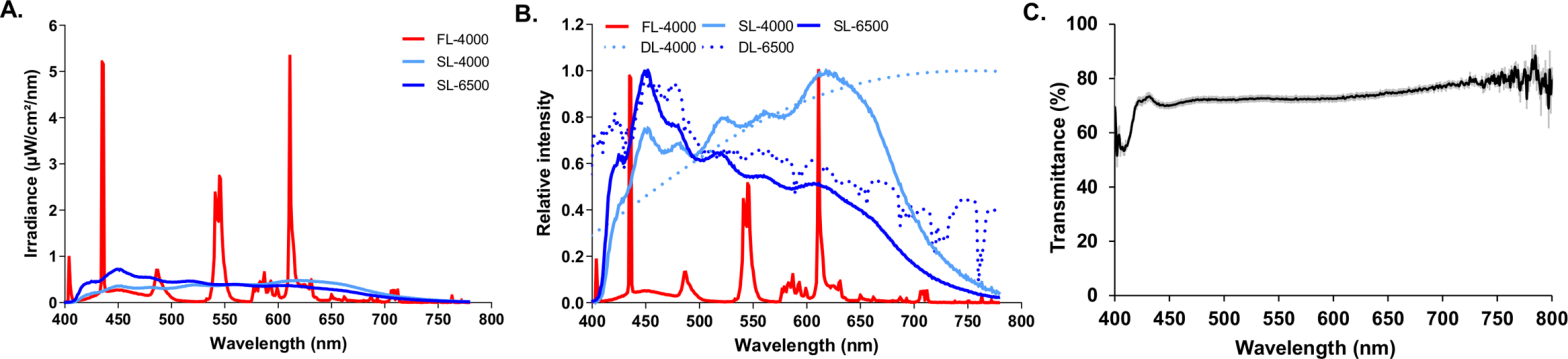
The absolute (**A**) and relative (**B**) spectral power distributions of fluorescent (4000 K: FL-4000) and full-spectrum LED (4000 K: SL-4000 and 6500 K: SL-6500) light-sources. Panel **B** also includes measurements of daylight/sunlight (4000 K: DL-4000 and 6500 K: DL-6500) for relative comparison of spectral power distribution between artificial and natural sunlight. The average (average ± SD) spectral light transmittance of 10 diffusers used in this study (**C**).

A total of 39, 1-day-old (D1) male Lohmann Brown chicks were obtained from a hatchery in Malaysia (Huat Lai Resources Berhad, Melaka, Malaysia) and were raised in a temperature-controlled enclosure (temperature maintained between 28°C and 32.5°C) with food and water ad libitum. The humidity of the dark room where the chicken enclosure was placed was maintained between 50% and 55%. Monocular FDM was induced over one randomly selected eye starting on D1 by placing a dome shaped 3D-printed translucent diffuser made of veroclear material (Stratasys, Eden Prairie, MN, USA) having a diameter of 13 mm, a thickness of 1.1 mm, and a radius of curvature of 7.5 mm. Different diffusers used for different animals yielded a very similar light spectral transmittance that was between 70% and 80% across the visible spectrum, except at the shortest wavelengths (<425 nm) where it was reduced to 50% to 70% (Fig. 1C). Diffusers were secured around the chicken's eye using Velcro rings, and were inspected twice daily during the 12-hour light period to ensure cleanliness. Contralateral eyes remained uncovered and served as controls.

Chicks were reared under a 12:12 hour light/dark cycle (0700–1900 hours) from day one (D1) until D28. On D1, chickens were divided into 3 groups based on the spectrum and CCT of isoluminant (approximately 285 lux) ambient lighting conditions: group FL-4000 (*n* = 18) was reared under fluorescent light (CCT = 4000 K, average illuminance ± SD: 281.8 ± 217.1 lux measured in all angles of gaze) with peaks in the spectral distribution of light at 435 nm, 545 nm, and 611 nm (T5 fluorescent tubes; OSRAM GmbH, Munich, Germany); group SL-4000 (*n* = 12) was reared under a full-spectrum Sunlike LEDs (SL LEDs; CCT = 4000 K, 284.5 ± 213.9 lux) with a continuous spectrum from 400 to 775 nm peaking at 618 nm (T5 Sunlike LEDs; Seoul Semiconductor Co. Ltd., Gyeonggi-do, South Korea); and Group SL-6500 (*n* = 9) was reared under a full-spectrum SL LEDs (CCT = 6500 K, 287.9 ± 219.9 lux) with a continuous spectrum from 400 to 775 nm peaking at 450 nm (T5 Sunlike LEDs; Seoul Semiconductor Co. Ltd.) (Figs. 1A, 1B). Additional light measurement details (e.g. chick lux, irradiance, and luminance) are provided in Supplementary Table S1. Light fixtures were controlled by a Helvar DIGIDIM 910 router (Helvar, Dartford Kent, UK). The spectral composition of the lights was assessed using a calibrated spectroradiometer ILT950 (International Light Technologies, Peabody, MA, USA). The spectral composition of daylight (6500 K) was measured using a calibrated spectroradiometer ILT950 (International Light Technologies) with the sensors facing upwards in an open field during at 12:00 PM on a cloudy day in Singapore. The spectral composition of daylight (4000 K) was provided by Seoul Semiconductor (Seoul Semiconductor Co. Ltd.) and mimics the spectrum of daylight at 7:00 AM in Seoul, South Korea (Fig. 1B). Average light intensities were calibrated at the chicken's eye level in all directions of gaze using a calibrated ILT 5000 radiometer (International Light Technologies). All animals used in this study were treated in accordance with the ARVO Statement for the Use of Animals in Ophthalmic and Vision Research. Protocols were approved by the SingHealth Institutional Animal Care and Use Committee (IACUC; AAALAC Accredited; 2018/SHS/1444).

### Ocular Measurements In Vivo

All ocular measurements were carried out between 12 PM and 5 PM and the animals were evaluated in a random order to minimize any circadian impact on the outcome measures. Non-cycloplegic refraction was assessed using an automated version of chicken infrared photoretinoscopy, as previously described by Schaeffel and Howland.^25^ Alert chicks were gently handheld on an adjustable platform approximately 1 meter away from the infrared photo-refractor. The chick's head was carefully positioned to ensure optimal focus on the chick's eye and detection of the first Purkinje image. Pupil size was adjusted using the operating software for each eye. The median of the most hyperopic refraction readings (i.e. resting refraction) with no accommodative changes was calculated from the continuous refraction trace over time in each animal's eye. Ocular AL, choroidal and retinal thicknesses, and anterior chamber depth (ACD) were measured in all animals on D1, D7, D14, D22, and D28 following protocols described by Najjar et al.^26^ The AL was defined as the distance between the echo spike corresponding to the anterior surface of the cornea and most anterior spike originating from the retina, and this was measured via A-scan ultrasonography using a PacScan Plus (Sonomed Escalon, New Hyde Park, NY, USA) at 10 MHz frequency. The median of at least five scans was calculated per eye for each animal. The average coefficient of variation for repeated AL measurements was 2.2 ± 1.0%. Choroidal and retinal thicknesses were measured using spectral-domain optical coherence tomography (SD-OCT; Spectralis; Heidelberg Engineering, Inc., Heidelberg, Germany). The average coefficient of variation for repeated choroidal measurements was 2.7 ± 1.6%, whereas the average coefficient of variation for repeated retinal measurements was 1.6 ± 0.8%. The ACD was calculated from scans of the anterior segment taken using anterior segment OCT (RTVue Optovue, Inc., Fremont, CA, USA). Baseline ocular parameters were measured on D1 before the application of the diffusers. During form-deprivation (D7 and D14), diffusers were removed for a brief period to perform ocular measurements. All measurements were carried out on non-anesthetized animals in a dimly lit room (<5 lux).

### Data Analysis

Results are presented as average ± standard deviation (SD). Changes in the measured parameters (AL, refraction, choroidal and retinal thicknesses, and ACD) of the form-deprived (FD) eye over the duration of the experimental procedure (D1 to D28) were compared with the control eyes (within each experimental group) then expressed as interocular differences (IODs) between the FD and the control eye (FD eye − control eye) and compared between experimental groups. After confirming normal distribution of the variables, ocular parameters and IODs were compared between eyes within the same group and among groups using a 2-way repeated-measures analysis of variance (2-way RM-ANOVA) with day and eye or day and group as within- and between-subject factors, respectively. Whole-body weights of animals in different groups were also compared using a 2-way RM-ANOVA. For those comparisons in which the omnibus test reached statistical significance, pairwise multiple comparison procedures were performed using the Holm-Sidak method. For all statistical tests, the level of significance was set at *P* < 0.05. Statistics were performed using Sigmaplot version 14.0 (Systat Software, Inc., San Jose, CA, USA), and plots were drawn using GraphPad Prism 6 (GraphPad Software, La Jolla, CA, USA).

## Results

### Characteristics of the Experimental Lights

Average illuminances, measured in all directions of gaze within the enclosure, were maintained at 281.8 lux (FL-4000), 284.5 lux (SL-4000), and 287.9 lux (SL-6500) throughout the experimental period. The relative spectra of SL LEDs (4000 K and 6500 K) were similar to that of outdoor sunlight/daylight (DL; 4000 K and 6500 K), being broader and more homogeneously distributed than that of FL-4000, which was discontinuous with sharp and spike-like peaks (see Figs. 1A, 1B). The total energy delivered at these peak wavelengths was much greater for FL-4000 than for the LED sources; however, FL-4000 delivered much higher energy than the LEDs near the peak spectral sensitivity of chicken UVS- and SWS-cones (ca. 420 and 455 nm, respectively), but energy comparable to that of the LEDs at the peak spectral sensitivity of LWS-cones (ca. 570 nm), and considerably less than that of the LEDs at the peak spectral sensitivity of MWS-cones (ca. 510 nm; see Fig. 1A).^27^ The overall average relative spectral transmittance of form-deprivation diffusers at the level of the chicken's eye was between 70% and 80%, except for wavelengths below 425 nm where it was decreased to a range of 50% to 70% (see Fig. 1C).

### Change in Body Weight of Experimental Groups

The average weights of the animals in the three groups were similar *(P* > 0.05) throughout the experimental protocol (Supplementary Fig. S1).

### Impact of Full Spectrum LEDs on Ocular Axial Length

**Figure 2.**
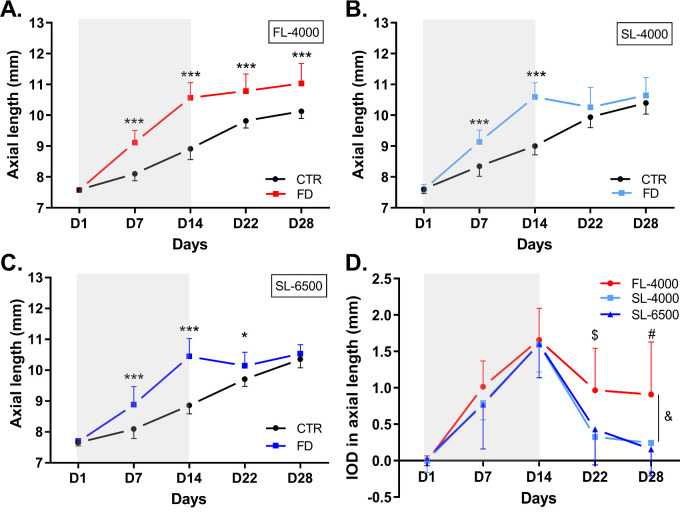
Axial length of FD and control eyes in animals raised under FL-4000 (*n* = 18) (**A**), SL-4000 (*n* = 12) (**B**), and SL-6500 (*n* = 9) (**C**); and interocular differences (IOD: form-deprived eye – control eye) in axial length of eyes in chicks exposed to FL-4000, SL-4000, and SL-6500 (**D**). Shaded area between D1 and D14 indicates the period of form-deprivation. &: Significant intergroup difference (*P =* 0.02), for FL-4000 versus both SL-4000 and SL-6500 (both: *P <* 0.05); $: at D22, IOD in axial length was significantly higher in FD eyes exposed to FL-4000 than in those exposed to SL-4000 *(P* < 0.001) or SL-6500 *(P* < 0.01); and #: at D28, IOD in axial length was significantly higher in FD eyes exposed to FL-4000 than in eyes exposed to SL-4000 (*P* < 0.001) or SL-6500 (*P* < 0.001). *(*P* < 0.05), ***(*P* < 0.001). CTR, control eyes; FD, form-deprived eyes. Data are represented as average ± SD.

Compared with control eyes, FD eyes showed an increase in AL on D7 and D14 in animals reared under FL-4000, SL-4000, and SL-6500 (all *P* < 0.001; Figs. 2A, 2B, 2C). Following the end of form-deprivation (D14), on D22 and D28, FD eyes exposed to SL-4000 and SL-6500 recovered more rapidly from excessive axial elongation than did FD eyes exposed to FL-4000 (see Figs. 2A, 2B, 2C). By D28, the differences between the AL of FD eyes in animals raised under SL-4000 (*P =* 0.14) and SL-6500 (*P =* 0.34), compared with control eyes, were no longer statistically significant (Figs. 2B, 2C); whereas in chicks reared under FL, the AL of recovered FD eyes was still 0.91 ± 0.72 mm longer than that of control eyes, on D28 (Figs. 2A, 2D, Table; *P* < 0.001).

**Table.** Comparison of Interocular Differences (Form-Deprived Eye – Control Eye) in Axial Length, Refraction, Choroidal Thickness, Retinal Thickness, and Anterior Chamber Depth in Animals Exposed to FL-4000, SL-4000, and SL-6500 Lights

		Days					*P* Values 2wRM-ANOVA		
Ocular Parameters	Condition	D1	D7	D14	D22	D28	Group	Day	Group x Day
**IOD (FD-CTR)**									
**Axial length** **,** **mm**	FL-4000^*,^ ^†^	−0.01 ± 0.08	1.01 ± 0.35	1.66 ± 0.44	0.97 ± 0.58	0.91 ± 0.72	0.02	<0.001	<0.001
	**SL-4000**	0 ± 0.16	0.79 ± 0.23	1.59 ± 0.37	0.32 ± 0.39^‡^	0.24 ± 0.39^‡^			
	**SL-6500**	0.03 ± 0.1	0.77 ± 0.61	1.6 ± 0.46	0.43 ± 0.49^§^	0.16 ± 0.36^‡^			
**Refraction** **,** **D**	**FL-4000**	0.01 ± 0.78	−9.82 ± 2.64	−12.9 ± 3.15	−8.15 ± 3.56	−5.84 ± 3.01	0.19	<0.001	0.49
	**SL-4000**	0.02 ± 0.98	−9.6 ± 1.75	−12.8 ± 3.81	−8.02 ± 3.98	−2.71 ± 4.81			
	**SL-6500**	−0.34 ± 2.89	−8.72 ± 4.49	−12.6 ± 2.68	−7.33 ± 3.57	−3.15 ± 2.87			
**Choroidal thickness, µm**	**FL-4000** ^∥^	−11.1 ± 31.5	−47.2 ± 50.4	−54.1 ± 36.4	239.0 ± 143.5	232.2 ± 138.0	0.03	<0.001	0.62
	**SL-4000**	9.58 ± 36.0	−61.9 ± 83.1	−57.3 ± 73.2	274.7 ± 93.6	282.3 ± 77.8			
	**SL-6500**	6.15 ± 21.6	−23.1 ± 37.5	14.9 ± 42.1	306.8 ± 39.5	299.2 ± 79.2			
**Retinal thickness, µm**	**FL-4000**	2.63 ± 17.1	−23.1 ± 17.7	−22.5 ± 27.3	−0.39 ± 25.8	−0.23 ± 18.6	0.22	<0.001	0.07
	**SL-4000**	−4.61 ± 11.7	−12.9 ± 26.1	−34.6 ± 26.2	5.47 ± 21.4	12.9 ± 29.8			
	**SL-6500**	−4.59 ± 12.1	−11.0 ± 26.0	−45.4 ± 19.7	−1.19 ± 22.1	−12.7 ± 20.5			
**ACD, mm**	**FL-4000**	0.02 ± 0.05	0.16 ± 0.13	0.5 ± 0.3	0.52 ± 0.38	0.32 ± 0.36	0.61	<0.001	0.85
	**SL-4000**	−0.01 ± 0.03	0.09 ± 0.08	0.42 ± 0.18	0.39 ± 0.21	0.27 ± 0.19			
	**SL-6500**	0.03 ± 0.03	0.08 ± 0.13	0.4 ± 0.19	0.33 ± 0.21	0.27 ± 0.26			

Data are presented as average ± SD. The *P* values represent the significance of the two way repeated measures ANOVA (2wRM-ANOVA) for “group,” “day,” and “group × day” comparisons. Post hoc pairwise comparisons using Holm-Sidak method are shown in this table for the factor “group” where ∥: FL-4000 is significantly different from SL-6500 (*P* < 0.05), and for the factor “group x day” where *: FL-4000 is significantly different from SL-4000; †: FL-4000 is significantly different from SL-6500 with respective significance levels § P < 0.01 and ‡ P < 0.001 indicated on the days of the experiment. CTR, control eye; FD, form-deprived eye.

The IOD in AL was significantly greater overall in the FL-4000 group than in the SL groups (*P =* 0.02); FL-4000 versus SL-4000 (*P =* 0.04), and FL-4000 versus SL-6500 (*P =* 0.049), but was not significantly different between the SL groups (see Fig. 2D, Table). This difference was dependent on the day of the experiment viz., on D22 and D28, the average IOD in AL of FD eyes exposed to SL-4000 and SL-6500 was significantly smaller than in eyes exposed to FL-4000 (see Fig. 2D, Table). The ALs of control eyes exposed to FL-4000, SL-4000, or SL-6500 were not significantly (*P* > 0.05) different throughout the experimental period (Supplementary Fig. S2A).

### Impact of Full Spectrum LEDs on Refraction

**Figure 3.**
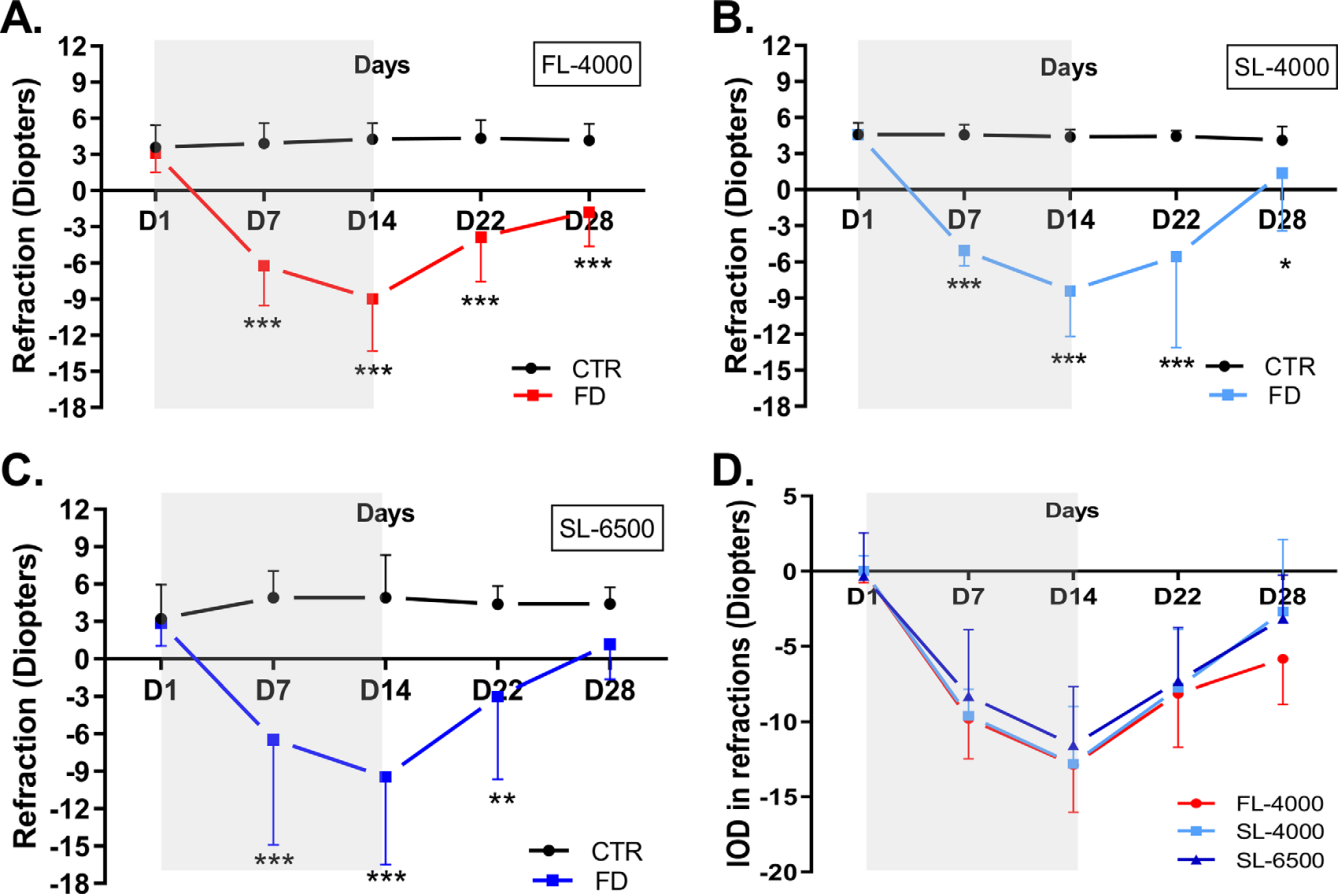
Refractive status of FD and fellow control eyes in animals raised under FL-4000 (*n* = 18) (**A**), SL-4000 (*n* = 12) (**B**), and SL-6500 (*n* = 9) (**C**), and interocular differences (IOD: form-deprived eye – control eye) in refractive status of eyes exposed to FL-4000, SL-4000, and SL-6500 (**D**). Statistical significance *(*P* < 0.05) **(*P* < 0.01), ***(*P* < 0.001). CTR, control eyes; FD, form-deprived eyes. Shaded area between D1 and D14 time points indicates the period of form deprivation. Data are represented as average ± SD.

In all groups, the spherical equivalent refractive error of FD eyes exhibited a significant myopic shift compared with control eyes (FL-4000: [*F* (1,34) = 244.44, *P* < 0.001]; SL-4000: [*F* (1,22) = 148.35, *P* < 0.001] and SL-6500; [*F* (1,16) = 29.44, *P* < 0.001]). This myopic shift predominantly took place during the form-deprivation period (D7 and D14; Figs. 3A–C). At the end of myopia recovery (D28), the refractions of FD eyes exposed to SL-6500 recovered to values similar to those of control eyes (see Fig. 3C; *P =* 0.14), whereas the refractions of FD eyes exposed to FL-4000 (see Fig. 3A; *P* < 0.001) or SL-4000 (see Fig. 3B; *P =* 0.04) remained significantly different from those of the control eyes. IODs in refraction among the three groups were not significantly different (see Fig. 3D, Table). The spherical equivalent refractions of fellow control eyes in chicks exposed to FL-4000, SL-4000, or SL-6500 were not significantly different (see Supplementary Fig. S2B).

### Impact of Full Spectrum LEDs on Choroidal and Retinal Thicknesses

**Figure 4.**
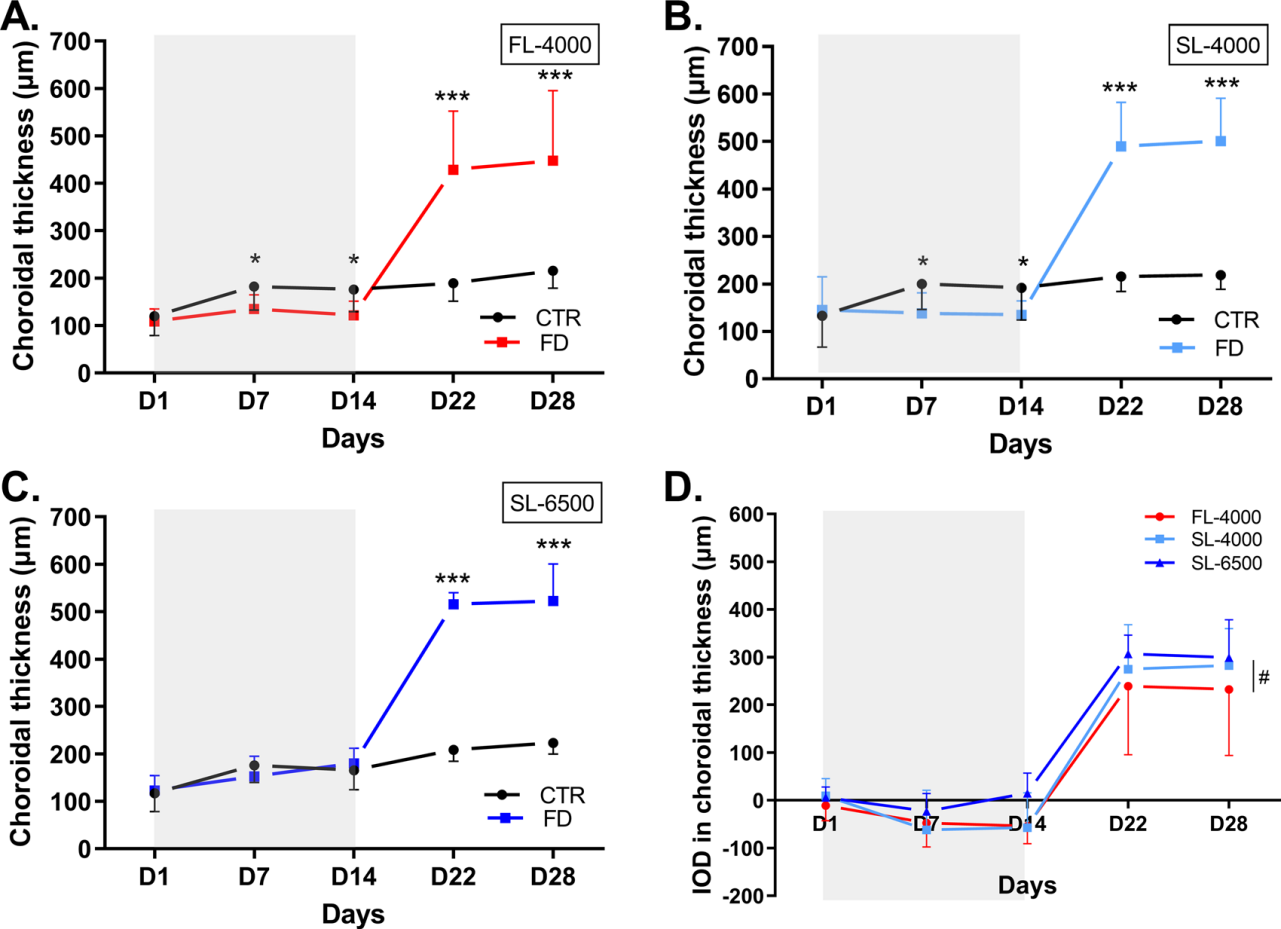
Changes in choroidal thickness of FD and fellow control eyes in animals raised under FL-4000 (*n* = 18) (**A**), SL-4000 (*n* = 12) (**B**), SL-6500 (*n* = 9) (**C**), and interocular differences (IOD: form-deprived eye – control eye) in choroidal thickness of eyes in chicks exposed to FL-4000, SL-4000, and SL-6500 (**D**). Statistical significance *** (*P* < 0.001); #: The IOD in choroidal thickness in FD eyes exposed to SL-6500 was significantly higher than in those exposed to FL-4000 (*P =* 0.02). CTR, control eyes; FD, form-deprived eyes. Shaded area between D1 and D14 indicates the period of form-deprivation. Data are represented as average ± SD.

In all groups, the choroidal thickness of FD eyes overall was different from that of fellow control eyes (FL-4000: [*F* (1,34) = 27.63, *P* < 0.001]; SL-4000: [*F* (1,22) = 76.52, *P* < 0.001] and SL-6500: [*F* (1,16) = 145.97, *P* < 0.001]; Figs. 4A, 4B, 4C). Choroidal thickness was significantly reduced in FD eyes compared with control eyes, on D7 ([FL-4000, *P =* 0.04]; [SL-4000, *P =* 0.02]) and D14 ([FL-4000, *P =* 0.02]; [SL-4000, *P =* 0.03]; see Figs. 4A, 4B); in FD eyes exposed to SL-6500, however, no significant choroidal thinning was observed on D7 and D14 of the form-deprivation period (Fig. 4C). During recovery from form-deprivation (i.e. D22 and D28), choroidal thickness increased in the previously FD eyes exposed to FL-4000, SL-4000, and SL-6500, compared with control eyes (all *P* < 0.001; see Figs. 4A–C). A significant group effect was found when comparing IOD in choroidal thickness (*F* (2,36) = 3.92, *P =* 0.03). Pairwise comparison revealed that the IOD in the choroidal thickness of the eyes exposed to SL-6500 was increased compared with eyes exposed to FL-4000 (*P =* 0.02; see the Fig. 4D, Table). IOD in choroidal thickness was not different between SL-4000 and SL-6500 (*P =* 0.2) or FL-4000 and SL-4000 (*P =* 0.3; Fig. 4D). Choroidal thicknesses of control eyes were not significantly different among groups (see Supplementary Fig. S2C).

The retina became significantly thinner by D14 in FD eyes compared to control eyes, in animals raised under FL-4000, SL-4000, and SL-6500 (FL-4000 [*P* < 0.001]; SL-4000 [*P* < 0.001]; and SL-6500 [*P* < 0.001]), whereas at D7, retinal thinning was found only in the FD eyes of animals raised under FL-4000 (*P* < 0.001; Supplementary Fig. S3). This effect was completely reversed by 8 to 14 days (D22 and D28, respectively) after diffuser removal, wherein the retinal thickness in FD eyes of all three groups was similar to that in control eyes (see Supplementary Fig. S3). IODs in retinal thickness in the three groups were not significantly different (see Supplementary Fig. S3D).

### Impact of Full Spectrum LEDs on Anterior Chamber Depth

**Figure 5.**
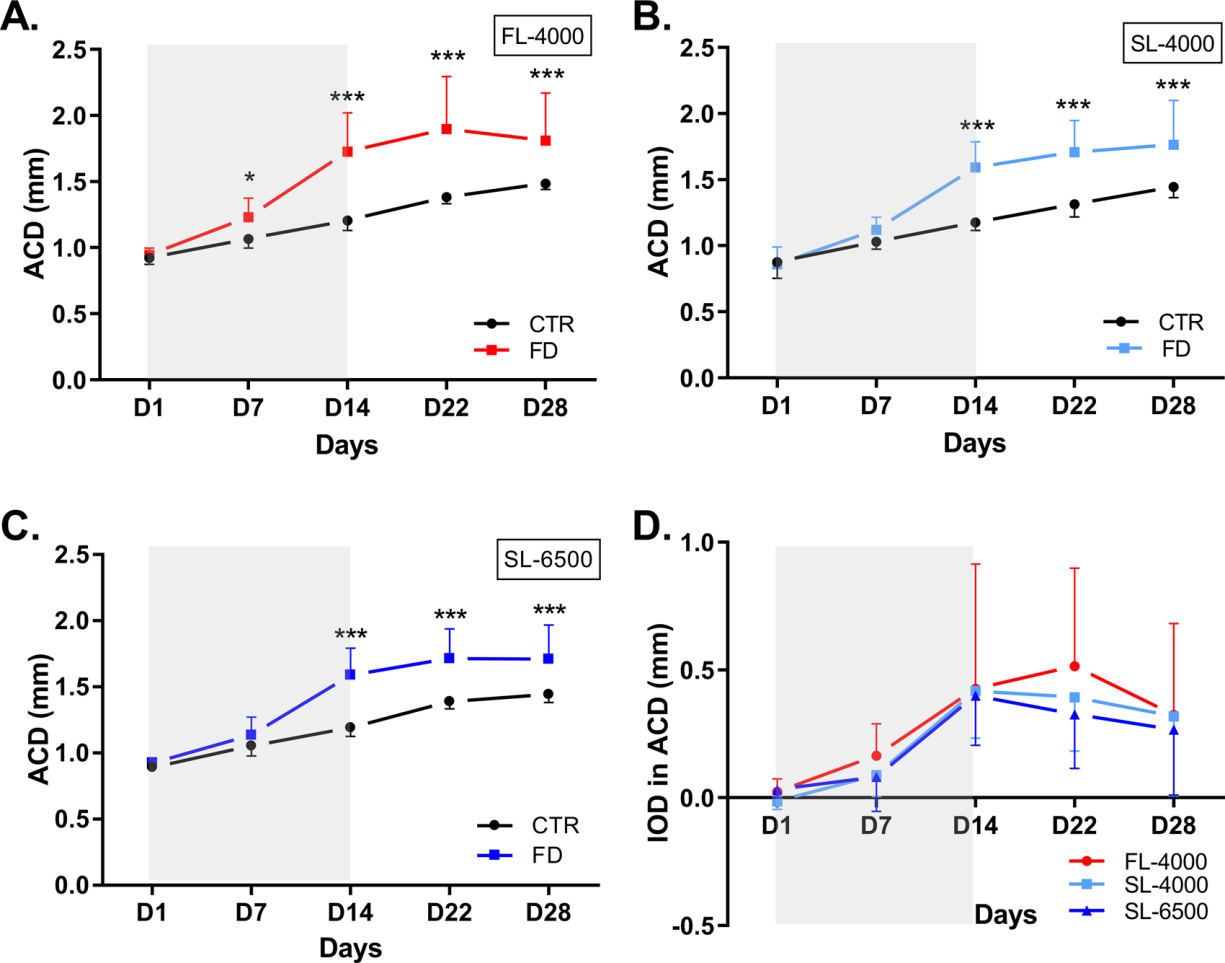
Changes in the anterior chamber depth of FD and fellow control eyes, in animals raised under FL-4000 (*n* = 18) (**A**), SL-4000 (*n* = 12) (**B**), SL-6500 (*n* = 9) (**C**); and interocular differences (IOD: form-deprived eye – control eye) in ACD of eyes exposed to FL-4000, SL-4000, and SL-6500 (**D**). Statistical significance ***(*P* < 0.001). ACD, anterior chamber depth; CTR, control eyes; FD, form-deprived eyes. Shaded area between D1 and D14 indicates the period of form-deprivation. Data are represented as average ± SD.

Under all lighting conditions, ACD was significantly greater in FD eyes than in control eyes (FL-4000: [*F* (1,34) = 24.56, *P* < 0.001]; SL-4000: [*F* (1,22) = 26.96, *P* < 0.001]; and SL-6500: [*F* (1,16) = 18.49, *P* < 0.001]; Figs. 5A, 5B, 5C). IODs in ACD in the three groups were not significantly (*P* > 0.05) different throughout the experimental period (see Fig. 5D). The ACD of control eyes were not significantly different among groups (Supplementary Fig. S2D).

## Discussion

In this study, we demonstrated that the spectral distribution and CCT of ambient “white” artificial light can affect the recovery from FDM in a chicken model. Irrespective of the CCT, both of the full-spectrum LED lights tested in this study (4000 K and 6500 K) accelerated recovery from the excessive axial elongation induced by form-deprivation, more completely than did isoluminant fluorescent light. None of the lights tested, however, halted the development of FDM, and only the higher-CCT continuous-spectrum LED light (6500 K) promoted a recovery from myopic refractive error to measures that were not significantly different from control eyes by the end of the experimental protocol. Furthermore, FD eyes of chicks reared under the higher-CCT continuous-spectrum LED light (6500 K) had thicker choroids than FD eyes exposed to fluorescent light, and did not exhibit any choroidal thinning on D7 and D14 of form-deprivation.

### The Impact of the Fullness of Spectrum on the Development of Form-Deprivation Myopia

Epidemiological investigations have highlighted the protective effect of spending time outdoors against human myopia.^10^ These findings were complemented by experimental research in animal models. For instance, Ashby et al.^28^ demonstrated that a diffuser-free exposure to 15 minutes of sunlight (approximately 30,000 lux) per day, significantly reduced the excessive increase in AL and myopic refraction induced by form-deprivation, when compared to a diffuser-free 15-minute exposures to normal (500 lux) or intense (15,000 lux) laboratory lights. Similarly, exposure of young rhesus monkeys to high-intensity sunlight (approximately 40,000 lux) inhibited the myopic shift induced by monocular hyperopic defocus (−3.0 D lenses).^29^ This protective effect against physiological and experimental myopia can potentially be attributed to the characteristics of sunlight, such as high intensity and full spectral compositions.^30^ Although many investigations have focused on the impact of intense light on experimental myopia development,^31^^–^^35^ only a few have investigated the impact of the spectral characteristics of ambient, moderate intensity, visible white light on refractive error development.^15^^,^^26^^,^^36^

In our study, Sunlike LEDs did not stop the development of FDM. These findings are in agreement with Li et al.,^15^ who reported no significant effect of full-spectrum halogen light on refractive error development in guinea pigs’ eyes having unrestricted vision compared with eyes having lens-induced myopia. Nevertheless, there are critical differences between our study and that of Li et al.^15^ For instance, our study used a FDM model, whereas the latter used lens-induced myopia. In addition, unlike Li et al.,^15^ we also investigated the effect of moderate levels of full-, continuous-spectrum light during the recovery phase, and the Sunlike LED spectra used in this study were different from that of Halogen light used by Li et al.^15^

### The Impact of the Fullness of Spectrum and CCT on the Recovery from Form-Deprivation Myopia

In our study, moderate levels of Sunlike LEDs (SL-6500 and SL-4000) accelerated recovery from FDM. These findings may reflect an improved emmetropization process under sunlike artificial light compared to florescent light, even when matched for CCT. These findings are in agreement with statements by Rucker et al.^37^^,^^38^ suggesting that broad spectrum light similar to sunlight, with a strong short-wavelength component, could enhance the emmetropization process. Ocular growth and emmetropization are dependent upon chromatic cues,^39^ and exposure to narrow-band blue light has been shown to induce hyperopia in some animal models (e.g. chickens^16^^,^^17^^,^^40^ and guinea pigs^18^^,^^41^) but not others (e.g. tree shrews^42^ and monkeys^21^^,^^43^). According to Rucker et al.,^38^ the spectral composition of broad-spectrum light might influence emmetropization through retinal mechanisms (circuitry) sensitive either to wavelength per se, or to wavelength-selective defocus due to longitudinal chromatic aberration (LCA). The LCA causes a higher refraction of short wavelength light compared to longer wavelength light by ocular structures, producing an additional chromatic cue for the sign of defocus determined by the eye.^39^ Concurrently, emmetropization is more accurate under polychromatic white light compared to monochromatic light^44^^,^^45^ and the emmetropization process can be facilitated by exposure to different visual environments, presenting visual stimuli over broad spectral and temporal ranges and rich in S-cone contrast.^38^ These are supposed to be the characteristics of an outdoor lighting environment, mimicked here by Sunlike LEDs. It is plausible that the fullness of the Sunlike light spectra (SL-4000 and SL-6500) compared to fluorescent light led to a broader detection of chromatic cues by the retina which led to a faster recovery from FDM. This hypothesis is further supported by the complete recovery from excessive axial elongation on D28 of the experiment under SL-4000 and SL-6500 lights. In addition, blue-enriched SL-6500 light, which yields a spectrum that is similar to sunlight around noontime (see Fig. 1B), promoted a recovery from myopic refractive error to measures that were not significantly different from control eyes on D28 of the experimental protocol. Previous reports have also shown that blue-enriched white light (CCT = 9700 K) can slow axial elongation induced by FDM, and accelerate recovery from FDM in a chicken model,^26^ whereas a recent study by Yoon et al.^36^ demonstrated that exposure to 985 lux of broad-spectrum light with a high blue content can slow axial elongation in chicks. Whether the effect of blue-enriched white light on recovery from experimental myopia is only due to LCA^46^ or other additional phenomena remains unclear. It is worth mentioning, however, that recovery from FDM may not closely mimic the process of emmetropization given the differences in ocular parameters (e.g. AL, choroid thickness, and ACD) between a control eye and a FD eye.

### Effect of Higher CCT on Choroidal Thickness

Choroidal thinning is a particular anatomical change related to myopia in humans^47^^,^^48^ and animal models.^36^^,^^49^ In our study, form-deprivation induced choroidal thinning in FD eyes exposed to fluorescent light and SL-4000. However, the choroid was thicker overall in FD eyes exposed to SL-6500 than in those exposed to fluorescent light, and there was no choroidal thinning under SL-6500 on D7 and D14 of form-deprivation. It remains plausible, however, that choroidal thinning may have occurred at an earlier stage of form-deprivation and was not captured by our measurements. Although it has already been reported that intense light (15,000 lux) induces choroidal thickening in chickens,^50^ here, we have shown that choroidal thickening in healthy and recovering FD eyes is dependent upon the spectral composition of white light. Recently, Najjar et al.^26^ showed that control eyes of chickens exposed to blue-enriched white light (CCT: 9700 K) showed thicker choroids when compared to the eyes exposed to soft white light (CCT: 3900 K). Similarly, FD eyes exposed to 9700 K light tended to have thicker choroids compared to FD eyes exposed to 3900 K light on D7 of form-deprivation. Nevertheless, under both lighting conditions, a marginal reduction in choroidal thickness was still observed on D14 of form-deprivation in FD eyes compared to the control eyes.^26^ Other authors have also suggested an impact of the spectral composition of light on choroidal thickness. Rucker and colleagues showed that the sinusoidal modulation of blue/yellow light with an intermediate temporal frequency of 5 Hz can reduce choroidal thinning in uncovered chicken eyes when compared to eyes exposed to red/green light.^37^ In humans, Lou and Ostrin demonstrated that choroidal thinning due to 1 hour exposure to red or darkness can be prevented with 1 hour exposure to narrow-band blue light.^51^ Taken together, these findings highlight a potential link between choroidal thickness and exposure to short wavelength light and suggest that, in our study, the absence of choroidal thinning during form-deprivation may be linked to the increased short wavelength content of SL-6500. However, this observation and whether the fullness of the light spectrum also contributes to choroidal thickness modulation deserves further dedicated investigations. After terminating form-deprivation, the choroids of recovering FD eyes were considerably thicker, irrespective of the lighting condition. This increase in choroidal thickness is considered as a compensatory mechanism for the resultant refractive error^49^ and has been attributed to changes such as the expansion of choroidal lacunae, increase in choroidal capillary permeability, proteoglycans production, aqueous humor outflow through uveo-scleral routes into the choroid, and decreased tone of the choroidal smooth muscle.^52^^,^^53^ As mentioned in the previous section of the discussion, recovery from FDM may not involve the same mechanisms as emmetropization and the marginally thicker choroids in the SL-6500 group may have led to a slower eye growth (faster recovery) as suggested by Nickla and Totonelly,^54^ where control eyes with thicker choroids grew slower than eyes with thinner choroids, however, this statement remains to be verified in the case of recovery from FDM.

### The Role of ACD in the Development and Recovery from Form-Deprivation Myopia

Aberrant axial elongation due to form-deprivation impacts the anterior segment of the eye. Although anterior segment changes have been observed in experimental myopia models, they are not directly related to the visual regulation of refractive state, but are rather epiphenomena of considerable changes in the posterior segment of the eye during form-deprivation.^55^ In our study, changes in ACD contributed to 29.5% or less in the increase in AL during the development of FDM, and 13.1% or less in the change in AL during the recovery from FDM (data not presented). Concurrently, the predominant contribution to changes in AL during the development and recovery from FDM came from changes in the posterior segment of the eye, more particularly the vitreous chamber (>70%). Troilo and colleagues suggested that changes in ACD are more of a programmed growth due to induced form-deprivation, whereas emmetropization involves visually guided process that enforces change in scleral growth and vitreous chamber depth.^24^ This is in agreement with our finding wherein the increased ACD did not revert back to normal during recovery from FDM.

### The Impact of CCT on the Untreated Control Eyes

The spectral composition of light, more specifically narrow-band light, can affect ocular growth and refractive development of uncovered control eyes.^16^^,^^17^^,^^46^ Furthermore, Yoon et al.^36^ reported that brighter broadband spectrum light around 1000 lux with increased blue content can reduce the axial elongation, whereas Najjar et al.^26^ reported a significant decrease in the AL, and increase in choroidal thickness of the control eyes exposed to blue enriched white lights with a CCT of 9700 K compared to 3900 K. The authors associated these observed changes with an increased blue content of the LED light. Such changes in the control eyes were not detected in our study. This is most likely due to the lower blue content and CCT of Sunlike LEDs (4000 K and 6500 K) compared to the 9700 K light used by Najjar et al.^26^ Our findings in the control eyes are also in agreement with Li et al.,^15^ wherein guinea pigs exposed to fluorescent and broad-spectrum white light exhibited similar refractive changes either under low (500 lux) fluorescent versus broad-spectrum or high (10,000 lux) fluorescent versus broad-spectrum light levels. Li et al.,^15^ however, did not specify the CCT of the experimental lights they used.

### Potential Pathways

One of the potential pathways for light-driven myopia-control is through retinal signaling molecules, acting via the retinal pigment epithelium and choroid to regulate scleral structure and biomechanics.^56^ In mammals, the sclera encompasses approximately 80% of the eye-wall and is recognized as the main load-bearing tissue of the eye; this is achieved through closely packed collagen fibers.^57^ Earlier studies on both humans and animals have shown that alterations to collagen micro-architecture and subsequent impact on biomechanical properties of sclera may also contribute to the myopia development.^58^^,^^59^ In addition, a recent meta-analysis of a genome wide association study comprising 160,420 participants of mixed ethnicity revealed 140 genetic associations linked with light-dependent pathways, which include genes involved in glutamate receptor signaling (*GNB3*) and dopaminergic actions (*DRD1*). These are involved in the light-dependent retina-to-sclera signaling cascade and subsequent visual regulation of ocular growth.^60^ Furthermore, Kusakari et al.^61^ demonstrated that diameter of the collagen fibers at the posterior pole of FD eyes were smaller when compared to controls. Taken together, it may be postulated that a change in scleral thickness in response to spectral component of light could also have attributed to the accelerated recovery. However, this requires further investigation to understand the exact mechanism.

### Limitations

Our study has a few limitations. First, we compared the impact of broad-, full-spectrum Sunlike LEDs to that of discontinuous-spectrum fluorescent light. Future studies should compare the effects of Sunlike LEDs to those of standard LEDs used in households. Second, owing to differences in the ocular systems of chicks and humans, our findings are not directly translatable to understanding and treating human myopia. Third, changes to the corneal curvature in response to FDM and different light exposures were not recorded in this study. These changes may have affected the refractive measurements and should be investigated in future studies. Further studies, benefitting from insights provided by non-primate animal studies such as ours, should investigate the impact of full-spectrum high CCT lighting on ocular growth in primate models.

## Conclusion

This study, using a chicken model of FDM, reveals that moderate levels of continuous-, full-spectrum LED lights that mimic sunlight can promote recovery from myopia and promote emmetropization after restoring normal viewing; and that full-spectrum light with higher CCT can prevent choroidal thinning that accompanies FDM on days 7 and 14. Our study supports previous reports showing that the spectral composition of indoor light could affect ocular growth and emmetropization, and open new research avenues for light-centered, passive myopia-control.

## Supplementary Material

Supplement 1
