# Supplementary material for: Recovery From Form-Deprivation Myopia in Chicks Is Dependent Upon the Fullness and Correlated Color Temperature of the Light Spectrum
Source: Invest Ophthalmol Vis Sci. 2022 Feb 8;63(2):16. doi: 10.1167/iovs.63.2.16 (PMC8822367; doi:10.1167/iovs.63.2.16)
Supplement: Supplement 1 [file iovs-63-2-16_s001.pdf]

Supplemental Material

Recovery from form-deprivation myopia in chicks is dependent upon the fullness and correlated color temperature of the light spectrum

Arumugam R. Muralidharan PhD[1,2], Low Wan Yu Shermaine BSc[1], Lee Yong Chong BVSc[1], Veluchamy A. Barathi PhD [1,2,3], Seang-Mei Saw MBBS, PhD [1,2,4], Dan Milea MD, PhD [1,2,5], Raymond P. Najjar PhD[1,2,3*]

[1] Singapore Eye Research Institute, Singapore; [2] Ophthalmology and Visual Sciences Academic Clinical Programme, Duke-NUS Medical School, Singapore; [3] Department of Ophthalmology, Yong Loo Lin School of Medicine, National University of Singapore, Singapore; [4] Saw Swee Hock School of Public Health, National University of Singapore, Singapore; [5] Singapore National Eye Centre, Singapore.

* Corresponding author

This supplement includes:

Supplementary Tables:

Supplementary Table 1. Detailed light measurements for experimental groups.

Supplementary Figures:

Supplementary Figure S1. Average weights of animals raised under FL-4000 (n = 18), SL-4000 (n=12) and SL-6500 (n = 9) throughout the experiment.

Supplementary Figure S2. Data from fellow control eyes in animals raised under FL 4000 (n=18), SL 4000 (n=12) and SL 6500 (n=9).

Supplementary Figure S3. Changes in the retinal thickness in FD and fellow control eyes in animals raised under FL-4000 (n = 18) (A), SL-4000 (n = 12) (B), SL-6500 (n = 9).

Supplementary References

This supplementary material has been provided by the authors to give readers additional information about their work**.**

Supplementary Tables

Supplementary Table 1. Detailed light measurements for experimental groups.

	**FL-4000**	**SL-4000**	**SL-6500**
**Illuminance (Human Lux)**	281.8	284.5	287.9
**Illuminance (Chick Lux)***	344.9	351.5	339.8
**Illuminance (Chick Lux)^**	423.6	444.2	510.1
**Irradiance (μW/cm²)**	92.2	107.5	118.4
**Luminance (cd/m²)**	126.2	110.9	128.0
**Log photon flux ($\log_{10}$ (1/cm²/s))**	14.4	14.5	14.5

** Photopic spectral sensitivity based on Nuboer et al., 1992 (ERG)*
^ Photopic spectral sensitivity based on Prescott and Wathes, 1999 (Behavioural)

Supplementary Figures

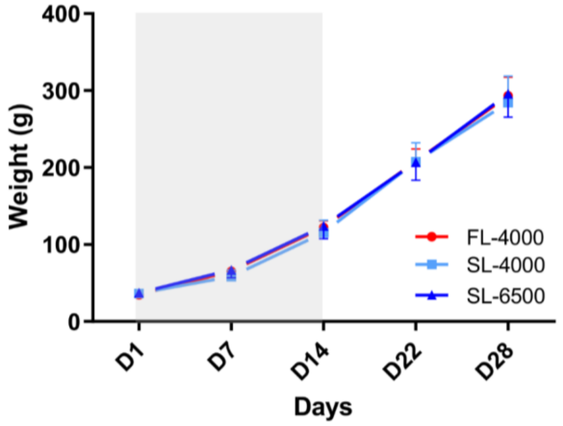

Supplementary Figure S1. Average weights of animals raised under FL-4000 (n = 18), SL-4000 (n=12) and SL-6500 (n = 9) throughout the experiment. Shaded area between D1 and D14 indicates the period of form-deprivation. Data are represented as average ± SD. Body weights were not significantly different between groups, and were not affected by form-deprivation or recovery (P>0.05).

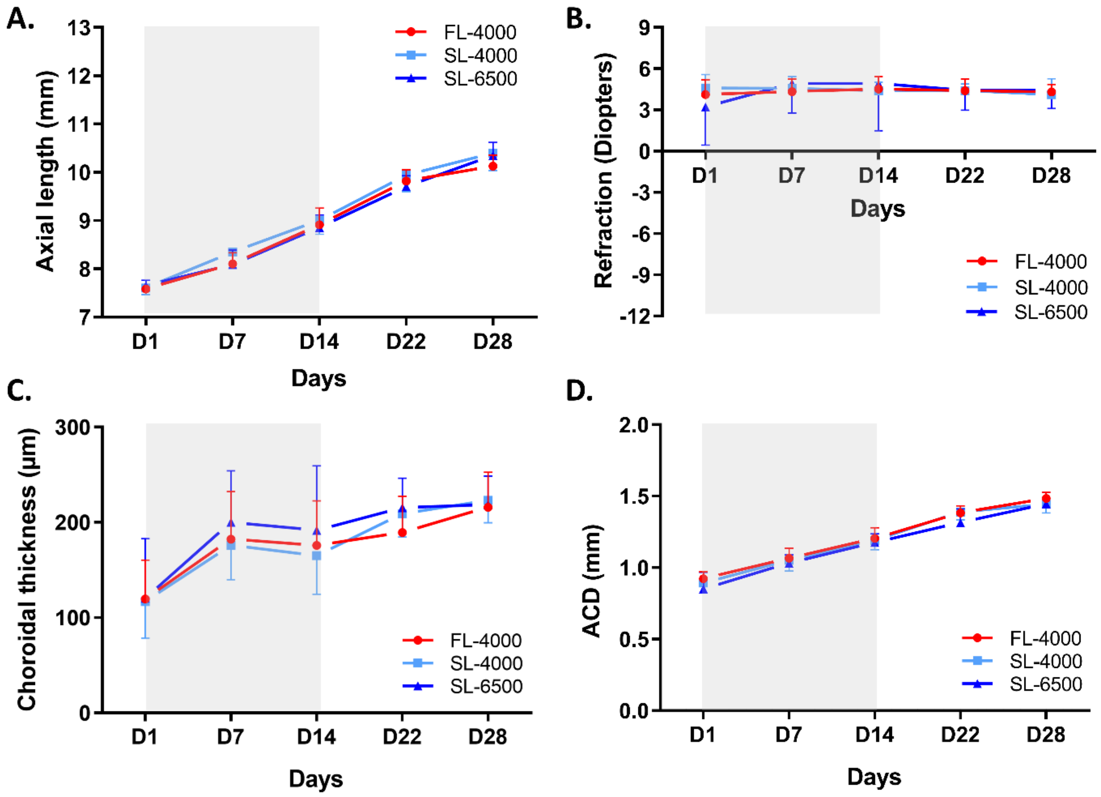

Supplementary Figure S2. Data from fellow control eyes in animals raised under FL 4000 (n=18), SL 4000 (n=12) and SL 6500 (n=9); axial length (A), refraction (B), choroidal thickness (C) and anterior chamber depth (ACD) (D). Data are represented as average ± SD. Data from control eyes were compared between groups using a 2-way repeated-measures analysis of variance. Shaded area (D1-D14) indicates the period of form-deprivation. No statistical differences were found between groups (P > 0.05).

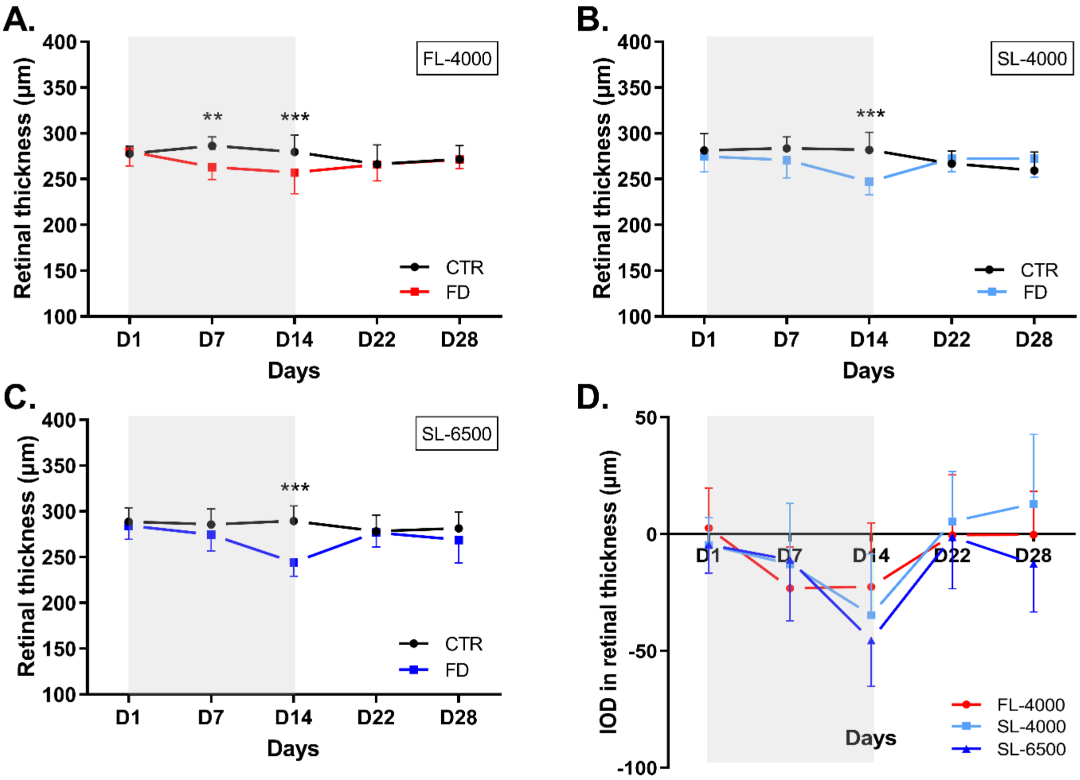

Supplementary Figure S3. Changes in the retinal thickness in FD and fellow control eyes in animals raised under FL-4000 (n = 18) (A), SL-4000 (n = 12) (B), SL-6500 (n = 9) (C); and interocular differences (IOD: form-deprived eye – control eye) in retinal thickness in eyes exposed to FL-4000, SL-4000 and SL-6500 (D). CTR: Control; FD: Form-deprivation. Shaded area (D1-D14) indicates the period of form-deprivation. Data are represented as average ± SD; ** (*P*<0.01), *** (*P*<0.001).

Supplementary References

1. Nuboer JFW, Coemans MAJM, Vos JJ. Artificial lighting in poultry houses: Are photometric units appropriate for describing illumination intensities?, Br Poult Sci. 1992; 33:1, 135-140.
2. Prescott NB, Wathes CM. Spectral sensitivity of the domestic fowl (Gallus g. domesticus). Br Poult Sci. 1999; 40: 332-9.
